# Implementation outcomes of the IMARA-South Africa mother-daughter HIV/STI prevention intervention: A mixed-methods study

**DOI:** 10.1017/cts.2025.10158

**Published:** 2025-09-23

**Authors:** Katherine G. Merrill, Millicent Atujuna, Saba Ahmed, Erin Emerson, Anelisiwe Ngcuka, Erin Jaworski, Linda-Gail Bekker, Natasha Crooks, Alyssa Debra, Geri Donenberg

**Affiliations:** 1 Center for Dissemination and Implementation Science, University of Illinois Chicago, Chicago, IL, USA; 2 Desmond Tutu HIV Centre, Institute of Infectious Disease and Molecular Medicine, University of Cape Town, Cape Town, South Africa; 3 College of Nursing, University of Illinois Chicago, Chicago, IL, USA; 4 College of Medicine, University of Illinois Chicago, Chicago, IL, USA

**Keywords:** Adolescent girls and young women, HIV prevention, implementation outcomes, implementation science, mother-daughter intervention

## Abstract

**Background:**

IMARA-South Africa (SA) is an HIV/STI prevention program for adolescent girls and young women (AGYW) and their female caregivers (FC). We examined six implementation outcomes of IMARA-SA (acceptability, appropriateness, feasibility, reach, adoption, and sustainability) from the perspectives of study staff, investigators, and collaborators.

**Methods:**

We used a sequential explanatory mixed-methods design. We administered surveys, hosted three focus group discussions with study staff/facilitators (*n* = 5), clinic staff (*n* = 3), and community advisory board members (*n* = 5), and conducted seven key informant interviews with investigators and study staff. We used descriptive statistics and rapid qualitative analyses, merging quantitative and qualitative data by implementation outcome to achieve triangulation.

**Results:**

On 27 surveys analyzed, mean scores were highest for acceptability (2.8/3, SD = 0.6), appropriateness (2.7/3, SD = 0.5), and reach (2.7/3, SD = 0.5), followed by feasibility (2.1/3, SD = 0.5), adoption (3.8/5, SD = 0.3), and sustainability (5.9/7, SD = 0.8). All perceived the AGYW and FC to love the program, which fit well with South African culture and addressed AGYW’s needs. The delivery site was deemed highly appropriate for reaching vulnerable populations. The lowest scoring items concerned time constraints (2.2/3, SD = 0.9), safety concerns (1.4/3, SD = 0.7), complexity (2.9/5, SD = 1.3), and cost (2.8/5, SD = 0.9). Qualitative participants attributed complexity and cost challenges to the research procedures, not the intervention. Participants proposed potential avenues for future implementation (e.g., schools, clinics) and interest in engaging males.

**Conclusion:**

IMARA-SA is implementable. Findings reveal challenges with navigating trade-offs between implementation outcomes and surveys distinguishing between intervention and research activities. Findings can inform future delivery of IMARA-SA and similar programs regionally.

## Introduction

Adolescent girls and young women (AGYW) in South Africa are disproportionately impacted by HIV. They account for 35% of new HIV infections in the country [1], and their incidence is three times higher than that of adolescent boys and young men [2]. Multiple social and structural factors drive high infection rates among AGYW, such as a high prevalence of age-disparate sex (estimated at 45% in a recent study) [3] and intimate partner violence (up to 45% in some regions of South Africa) [4,5]. There is increasing recognition that reducing HIV incidence among AGYW requires prevention approaches that tackle these social and structural determinants of risk, going beyond traditional biomedical approaches [6].

IMARA-SA (Informed, Motivated, Aware, and Responsible Adolescents and Adults- South Africa) is a culturally tailored intervention for AGYW and their female caregivers (FC) (e.g., mothers, sisters, aunts, grandmothers) designed to reduce HIV-related disparities by empowering AGYW with sexual risk prevention skills [7]. The intervention draws on a relatively underutilized resource for HIV prevention in South Africa – namely, the engagement of families, which play a central role in the growth and sexual decision-making of AGYW [8,9]. Some studies have sought to engage parents in HIV prevention [10–12] and to support youth living with HIV [13–15]. However, we are unaware of other programs specifically engaging AGYW and their FC in HIV/STI prevention in the region despite the important part that mothers, in particular, play in promoting safer sexual behavior of their adolescents [16]. Our team found promising early findings on the intervention’s reach, feasibility, acceptability, fidelity, and effectiveness in a pilot study with 59 AGYW-FC dyads [17]. A randomized controlled trial (RCT) was subsequently conducted to assess the impact of IMARA-SA on incident sexually transmitted infections (STIs), HIV testing and counseling (HTC), and pre-exposure prophylaxis (PrEP) uptake [7]. Analyses of the RCT data (currently in progress) will shed light on the intervention’s effectiveness in achieving its desired health outcomes.

Beyond the assessment of intervention health outcomes, there is growing recognition of the need to bridge the gap between research and practice by scientifically studying questions about implementation [18]. Implementation outcomes – i.e., the effects of deliberate actions to implement an intervention [19] – are key ingredients in implementation success. They are necessary precursors to desired health outcomes, given that an intervention cannot achieve effectiveness if not implemented well [19]. Proctor et al. proposed a taxonomy of eight implementation outcomes [19], comprising the implementation outcomes framework, which sparked notable advancements in the implementation science field [20]. Increasingly, researchers are assessing effectiveness and implementation outcomes within the same study through hybrid effectiveness-implementation designs [21]. Concurrent examination of both types of outcomes can accelerate the speed at which research findings are put into practice [22]. The literature examining implementation experiences of HIV prevention programming for AGYW in the region is growing [23,24], but there remains a need for further understanding of the implementation outcomes to strengthen program delivery and increase the potential for health impacts.

This study examined the implementation outcomes of IMARA-SA as a sub-study within the ongoing RCT. We focused on the perspectives of IMARA-SA study staff, investigators, and collaborators. Study staff and other individuals supporting delivery are well-suited to sharing perspectives on implementation outcomes, given their unique views on organizational and systems factors that affect delivery. Our study aim was to examine six implementation outcomes of IMARA-SA – i.e., acceptability, appropriateness, feasibility, reach, adoption, and sustainability – from the perspective of IMARA-SA staff, investigators, and collaborators, drawing on the implementation outcomes framework [19].

## Materials and methods

### Study design

This study used an sequential explanatory mixed methods design. Quantitative data were collected first, followed by qualitative data, with equal value placed on each to value the strengths and limitations of each method (i.e., QUANT → QUAL) [25,26]. Both types of methods were used to address the same study aim (i.e., a convergence function) [26].

### Parent study

This study was a sub-study within a larger RCT conducted with AGYW (15–19 years old) and their FC (24+ years) from Phillippi and surrounding townships (e.g., Nyanga, Gugulethu, Khayelitsha) in the Cape Town metropolitan area (details reported elsewhere [7]). Dyads of AGYW and their FC were recruited through street outreach, word-of-mouth referral, flyers, clinic contacts by study personnel, and walk-ins to the clinic. They were randomized 1:1 to the IMARA-SA treatment arm or a health promotion control group. Female interventionists of similar backgrounds to the participants delivered the intervention (roughly 10 hours long) over two days at the DTHF offices in Philippi Village. Participants completed assessments – including surveys, STI testing, HIV testing, and counseling – at baseline, 6 months, and 12 months. At the end of each workshop day, participants also completed surveys about their perceptions of the acceptability, appropriateness, and feasibility of the intervention (findings to be reported elsewhere). Participants were reimbursed 250 rand cash or the equivalent in vouchers/gift cards for each study visit.

### Positionality statement

The study was carried out through a collaboration between the Desmond Tutu Health Foundation (DTHF) in South Africa and the Center for Dissemination and Implementation Science in Health Disparities (CDIS) at the University of Illinois Chicago (UIC). Our authorship team includes 10 individuals affiliated with DTHF and UIC. All co-authors identify as female – aligning with the focus of the intervention on women and girls – and contributed expertise in HIV/STI risk among AGYW in South Africa, family-based interventions, implementation science, and mixed-methods studies. While carrying out this study, we acknowledged how our identities, nationalities, and backgrounds shaped our approach to the research and our interpretation of findings. We used extensive discussion and analytical memos [27] to critically reflect on our positionalities and ensure that our findings would achieve goodness – i.e., where emerging themes authentically represent the data [28].

### Intervention overview

IMARA-SA is an HIV/STI prevention intervention for AGYW and their FC, adapted from the evidence-based IMARA program for Black adolescent girls and their mothers in the U.S. [29,30]. AGYW and FC participate in separate and joint activities, which are interactive and experiential, over two days. The curriculum addresses individual factors (e.g., knowledge and attitudes about HIV/STIs), social factors (e.g., the family context), and structural factors (e.g., gender dynamics) that drive HIV risk (additional detail is presented elsewhere [7]). The program aims to enhance the credibility of the FC as a resource for AGYW in HIV/STI prevention. The group format is designed to facilitate structural change by building community norms for practicing prevention (e.g., HIV testing and counseling and PrEP uptake) and reducing HIV stigma. The goals and motto emphasize strong relationships between AGYW and FCs, as well as sisterhood, empowerment, and HIV/STI prevention motivation. Participants are given homework at the end of Day 1 to complete before Day 2 to facilitate reflection on learnings.

### Participants and procedures

Data were collected between 30/8/2021 and 11/4/2024. Individuals were eligible for the study if they were a study staff member, investigator, or collaborator on the IMARA-SA study or if they were a staff member or administrator from DTHF, 18 years or older, and spoke English. Each group offered different but valuable perspectives on the project. IMARA-SA study staff were directly involved in the day-to-day running of the study. Investigators were either directly supporting the study’s implementation or heavily involved in decision-making throughout the study through bi-weekly calls hosted between the SA and US teams. “Collaborators” included individuals who were not part of the study team but had advised on the conception and/or implementation of IMARA-SA. Finally, DTHF staff or administrators were less intimately involved but were regularly informed about the project and thus well-positioned to offer insights into how the project fit into the landscape of DTHF and other similar programs in the area.

We invited all South Africa-based IMARA-SA study staff to complete surveys. We also invited select DTHF finance and grants staff, as well as administrators, who were involved in implementing study budgets and procuring project resources. Baseline surveys were administered prior to the start of the RCT’s launch or when joining the study (for study staff). Follow-up surveys were administered roughly 18 months after baseline surveys or prior to a study staff member transition off the study (mean = 14.3 months, SD = 6.2 months). No reimbursement was given for surveys completed. This study reports on demographic data collected at baseline and implementation outcome data collected at follow-up.

About 2.5 years after the RCT’s launch, we conducted three focus group discussions and seven key informant interviews with study team members in South Africa and the U.S., using the same eligibility criteria. All invited individuals from the IMARA-SA study team in South Africa had completed the quantitative surveys except for one; U.S.-based IMARA-SA study team members had not been invited to complete the quantitative surveys. The first focus group was conducted with IMARA-SA study staff (*n* = 5), including facilitators of the IMARA-SA program and those involved in recruiting and transporting participants. A second focus group was conducted with clinical staff who conducted clinical assessments for the IMARA-SA study (i.e., HIV and STI testing and administration of PrEP) (*n* = 3). A third focus group was conducted with members of the DTHF Community Advisory Board (CAB) (*n* = 5), whose input on the project was first sought over five years ago. The CAB members were intimately familiar with the project, having provided input on the adaptation and testing of IMARA-SA through both the piloting and RCT phases. Focus groups were hosted in person at DTHF offices and lasted about 90 minutes. Key informant interviews (*n* = 7) were conducted with the study co-principal investigators (co-PIs), co-investigators, SA and U.S.-based project directors, and the study physician. Interviews were hosted in person or over Zoom and lasted about 30 minutes. CAB members were reimbursed 200 rand for their time and transport for participating in a focus group discussion. No reimbursement was given for study or clinic staff participating in focus groups or for key informants.

KM moderated the focus groups with IMARA-SA study and clinic staff and led the key informant interviews in English. KM and MA co-moderated the focus group with CAB members, which was hosted in English and Xhosa with simultaneous translation by MA. Focus groups and interviews were audio-recorded and transcribed verbatim. KM wrote analytical memos after focus groups and interviews to capture reflections on the data collection tools, interpret preliminary findings, and document the research process [27,28].

### Frameworks and outcomes

Proctor’s implementation outcomes framework [19] informed our decision to examine six implementation outcomes: acceptability, appropriateness, feasibility, reach, adoption, and sustainability (Table 1). Data were also collected on fidelity and will be reported elsewhere.

**Table 1.** Implementation outcomes examined, with definitions

Outcome	Definition
Acceptability	The perception that IMARA-SA is agreeable, palatable, or satisfactory
Appropriateness	The perceived fit, relevance, or compatibility of IMARA-SA for HIV/STI prevention in South Africa
Feasibility	The extent to which IMARA-SA can be successfully carried out in the delivery setting
Reach	The number, proportion, and representativeness of individuals willing to participate in IMARA-SA [31]*
Adoption	The intention, initial decision, or action to employ IMARA-SA (i.e., “uptake”)
Sustainability	The extent to which IMARA-SA is maintained or institutionalized

*Note: we drew on Glasgow et al.’s definition for reach [31] instead of Proctor’s outcome of penetration [19], given its greater applicability to delivery in a community versus service setting.

### Quantitative measures and qualitative guides

Surveys included demographic questions and a question about the respondent’s familiarity with the IMARA-SA curriculum. Participants completed items from the mental health implementation science tools (mhIST), a valid and reliable instrument of implementation outcomes used across diverse populations in low- and middle-income countries [32]. To maintain a reasonable survey length, we selected five of the most relevant items from the acceptability sub-scale (the sample size was too small to generate a Cronbach’s alpha), appropriateness sub-scale (Cronbach’s alpha = 0.93), feasibility subscale (Cronbach’s alpha = 0.68), and reach sub-scale (Cronbach’s alpha = 0.95) of the mhIST. We also included 11 items from the reliable Antiretroviral Treatment Access Study (ARTAS) [33] to measure the likelihood of adoption (Cronbach’s alpha = 0.79). Finally, we selected 17 items from the valid and reliable Program Sustainability Assessment Tool (PSAT) [34] to measure sustainability, including 5 items measuring environmental support, 3 items measuring partnerships, 5 items measuring organizational capacity, and 4 items measuring strategic planning (Cronbach’s alpha = 0.94).

Focus group and interview guides were tailored to participants and included open-ended questions prompting participants to expand on the implementation outcomes addressed in questionnaires (e.g., “What did you like about the IMARA sessions? What did you dislike about the sessions?” “To what extent did the recruitment team reach adolescent girls and their caregivers who could benefit from the program?”). The focus group with IMARA-SA facilitators probed further into views on the curriculum, while the key informant interviews with investigators probed more into perceptions of adoption and potential sustainability.

### Analyses

Quantitative data were analyzed using descriptive statistics (e.g., means, standard deviations). To support interpretation, we generated a combined mean for several items from the same scale that addressed similar topics – i.e., for safety in the feasibility scale and financial implications in the adoption scale. We ran t-tests to test if mean differences across the outcomes varied by whether an individual had been supporting IMARA for less than one year compared to one year or more.

Qualitative data were analyzed using a rapid analysis approach [35,36]. A framework matrix was generated, based on the focus group and interview guides [35,37]. SA extracted data into the framework matrix, which was checked for accuracy by KM. Emerging themes were identified through extensive discussions between KM, SA, and MA and additional discussion with the full authorship team. We used data triangulation, merging the quantitative and qualitative data by implementation outcome [26,28]. During this process, quantitative and qualitative methods were equally valued, and both consistencies and inconsistencies across the methods were identified. Our goal was for completeness [28], where the possibility of multiple realities was recognized in an effort to generate a holistic understanding of findings.

### Ethical considerations

Written informed consent was obtained from all study participants. This research was approved by the Institutional Review Boards of the University of Cape Town (077/2019) and the University of Illinois Chicago (2018–0709).

## Results

### Participant characteristics and overall trends

Surveys were completed by 27 staff and collaborators (Table 2). Half of study staff (non-facilitators) (50%, *n* = 9), all of IMARA facilitators (*n* = 5), and 25% of DTHF staff/administrators (*n* = 1) reported knowing the curriculum “very well” with the remainder reporting knowing the curriculum “somewhat well” or “not well.”

**Table 2.** Characteristics of survey participants (*n* = 27)

Variable	*n* (%)
**Gender**	
Female	22 (81.5%)
Male	5 (18.5%)
**Age group**	
24–29 years	8 (29.6%)
30–39 years	9 (33.3%)
40–49 years	8 (29.6%)
50+ years	2 (7.4%)
**Race**	
Black	20 (74.1%)
White	3 (11.1%)
Colored	2 (7.4%)
Indian	2 (7.4%)
**Main language spoken at home**	
Afrikaans	1 (3.7%)
English	9 (33.3%)
Xhosa	13 (48.2%)
Zulu	1 (3.7%)
Setswana	2 (7.4%)
Songa	1 (3.7%)
**Highest level of education completed**	
St 10 / Grade 12 Matric	5 (18.5%)
Post Matric / Diploma / Courses	4 (14.8%)
Graduate degree	11 (40.7%)
Post Graduate	7 (25.9%)
**Length of time supporting IMARA-SA**	
Less than one month	4 (14.8%)
One to 5 months	8 (29.6%)
Over 5 months to less than one year	4 (14.8%)
One year to less than 2 years	3 (11.1%)
Two yeas or more	8 (29.6%)

Mean scores were highest for acceptability, appropriateness, and reach, followed by feasibility, adoption, and sustainability. Across quantitative findings, mean scores were highest among IMARA-SA staff (non-facilitators) and IMARA-SA facilitators compared to DTHF staff and administrators. We did not find evidence that mean scores across outcomes different by whether participants had supported IMARA for less than a year compared to one year or more. We proceed to unpack these results by implementation outcome.

### Acceptability

Acceptability scores were high across roles, averaging 2.8/3.0 (SD = 0.6) (Table 3). Focus group participants and interviewees alike reported that AGYW and FC loved the program. The clinical staff focus group depicted participants as “feeling happy” and “laughing” during and after sessions. Focus group participants especially liked the program’s inclusion of separate and joint sessions for the AGYW and FC to give protected time for open discussion in each group, as well as the flexibility for AGYW to choose which caregiver they wanted to invite to the workshop. A participant from the CAB focus group said of IMARA-SA, *“I wish it could continue for a lifetime.”*

**Table 3.** Ratings of acceptability, appropriateness, feasibility, and reach in the full sample and by role

		Total		IMARA-SA study staff (non- facilitators)		IMARA-SA study staff (facilitators)		DTHF staff/ administrator	
#	**Acceptability** *(scale of 0-3, where 0=“not at all” and 3=“a lot”)*	*n*	Mean (SD)	*n*	Mean (SD)	*n*	Mean (SD)	*n*	Mean (SD)
1	Overall, do you like delivering IMARA?	5	3.0 (0.0)	–	–	5	3.0 (0)	–	–
2	Do you feel like the skills you learned by delivering IMARA are useful?	5	3.0 (0.0)	–	–	5	3.0 (0)	–	–
3	In general, did you feel comfortable answering participants’ questions?	5	2.8 (0.4)	–	–	5	2.8 (0.4)	–	–
4	Were you satisfied with your abilities delivering IMARA?	5	3.0 (0.0)	–	–	5	3.0 (0)	–	–
5	Overall, do you like IMARA?	27	2.8 (0.6)	18	2.8 (0.5)	5	3.0 (0)	4	2.5 (1.0)
	**Total**	**27**	**2.8 (0.6)**	**18**	**2.8 (0.5)**	**5**	**2.96 (0.1)**	**4**	**2.5 (1.0)**
#	**Appropriateness** *(scale of 0-3, where 0=“not at all” and 3=“a lot”)*	*n*	Mean (SD)	*n*	Mean (SD)	*n*	Mean (SD)	*n*	Mean (SD)
1	How well does the IMARA intervention fit with your cultural values?	27	2.6 (0.7)	18	2.6 (0.7)	5	3.0 (0.0)	4	2.0 (0.8)
2	How well does the IMARA intervention fit with your personal values?	27	2.7 (0.5)	18	2.8 (0.4)	5	3.0 (0.0)	4	2.0 (0.8)
3	How well does the IMARA intervention fit with the female culture in South Africa?	27	2.6 (0.6)	18	2.7 (0.5)	5	3.0 (0.0)	4	1.8 (0.5)
4	Do you feel the Desmond Tutu Health Foundation/Philippi Village is a good place for participants to attend IMARA?	27	2.9 (0.5)	18	2.9 (0.2)	5	3.0 (0.0)	4	2.3 (1.0)
5	Do you think the IMARA intervention addresses problems that are common in Klipfontein/ Mitchells Plain & neighboring areas?	27	2.8 (0.5)	18	2.9 (0.2)	5	3.0 (0.0)	4	1.8 (0.5)
	**Total**	**27**	**2.7 (0.5)**	**18**	**2.8 (0.3)**	**5**	**3.0 (0.0)**	**4**	**2.0 (0.7)**
#	**Feasibility** *(scale of 0-3, where 0=“not at all” and 3=“a lot”)*	*n*	Mean (SD)	*n*	Mean (SD)	*n*	Mean (SD)	*n*	Mean (SD)
1	In general, was there enough time to deliver the IMARA intervention?	23	2.2 (0.9)	18	2.2 (0.9)	5	2.2 (0.5)	–	–
2	How safe did you feel traveling to the place where the IMARA intervention was delivered?	23	1.4 (0.7)	18	1.3 (0.8)	5	1.8 (0.5)	–	–
3	In general, how safe is the place where the IMARA intervention was delivered?	23	1.3 (0.9)	18	1.2 (1.0)	5	2 (0)	–	–
4	In general, do you feel the place where the IMARA intervention was delivered was confidential?	23	2.7 (0.5)	18	2.7 (0.6)	5	3 (0)	–	–
5	Do you believe women in Klipfontein/ Mitchells Plain could seek HIV care from IMARA w/out fear of how others would view them?	27	2.6 (0.6)	18	2.7 (0.6)	5	3 (0)	4	2.0 (0.8)
	**Total**	**27**	**2.1 (0.5)**	**18**	**2.0 (0.5)**	**5**	**2.4 (0.6)**	**4**	**2.0 (0.8)**
#	**Reach** *(scale of 0-3, where 0=“not at all” and 3=“a lot”)*	*n*	Mean (SD)	*n*	Mean (SD)	*n*	Mean (SD)	*n*	Mean (SD)
1	Were people in Klipfontein/ Mitchells Plain aware that IMARA was available?	19	2.5 (0.7)	11	2.5 (0.7)	4	2.8 (0.5)	4	2.3 (1.0)
2	Did most women in Klipfontein/ Mitchells Plain who needed HIV prevention agree to participate in IMARA when invited?	20	2.7 (0.7)	12	2.8 (0.6)	5	3.0 (0)	3	2.0 (1.0)
3	Did the poorest women in Klipfontein/ Mitchells Plain who needed HIV prevention agree to participate in IMARA when invited?	18	2.8 (0.5)	11	2.9 (0.3)	5	3.0 (0)	2	2.0 (1.4)
4	Did most female caregivers in Klipfontein/ Mitchells Plain who needed HIV prevention agree to participate in IMARA when invited?	23	2.7 (0.5)	14	2.9 (0.4)	5	3.0 (0)	4	2.0 (0.8)
5	Did female caregivers in Klipfontein/ Mitchells Plain agree to participate in IMARA when invited if their daughters needed HIV prevention?	22	2.8 (0.5)	14	2.9 (0.3)	5	3.0 (0)	3	2.0 (1.0)
	**Total**	**25**	**2.7 (0.5)**	**14**	**2.9 (0.3)**	**5**	**2.96 (0.1)**	**4**	**2.1 (0.8)**

Abbreviations: IMARA = IMARA-South Africa.

Note: Items were adapted from the mental health implementation science tools (mhIST) [32].

Scores were similarly high among IMARA-SA facilitators regarding the extent to which they liked and felt satisfied with the intervention and its delivery, the skills they learned, and their comfort answering participants’ questions (2.96/3.0, SD = 0.1). In their focus group discussion, IMARA-SA facilitators and study staff described five key components of the intervention based on their involvement with the adaptation of IMARA to a South African context: 1) basic information about STIs and HIV (e.g., a poster of common STIs, activities on risky behavior); 2) how to use condoms; 3) assertive communication between mothers and daughters; 4) relationship content addressing healthy versus unhealthy relationships, gender-based violence, and partner communication; and 5) content on self-esteem and culture, including the integration of poems and songs. These participants viewed these components of the intervention as highly acceptable and essential to the intervention.

### Appropriateness

Appropriateness scores were also high across roles, averaging 2.7/3.0 overall (SD = 0.5) and 3.0/3.0 among IMARA-SA facilitators (Table 3). Based on survey responses, IMARA-SA was perceived to fit well with respondents’ cultural values, personal values, and the female culture in South Africa and was thought to address problems common in the area. Interviewees who were part of the two-year adaptation and piloting of IMARA-SA spoke about the systematic process through which the intervention was adapted to ensure its appropriateness among South African families. One interviewee described how the curriculum manages to walk a fine line between encouraging AGYW to express their needs and wants to their FC without undermining FC: *“We tried to make sure that the curriculum can empower the mother but also enable the child to communicate well so that the two can build a good relationship.”*

Scores were similarly high for perceptions that the site where IMARA-SA was delivered is a good place for participants to attend the program (2.9/3.0, SD = 0.5). Qualitative findings underscored the critical importance of delivering IMARA-SA in a community location accessible to the population of interest. The delivery site was thus perceived as highly appropriate for achieving reach (see further details below). An interviewee explained the reasons for why a community location was so critical: *“It is a very, very vulnerable community here. We have very high STI rates, and the HIV rate is high.”*

### Feasibility

On the feasibility scale, scores were high across roles for the confidentiality of the space where IMARA-SA was delivered (2.7/3.0, SD = 0.5) and the perception that women in the community could seek HIV care from IMARA-SA without fear of how others would view them (2.6/3.0, SD = 0.6) (Table 3). Qualitative findings on recruitment and retention were positive. Recruitment conducted through DTHF community officers, non-governmental organizations (NGOs), other DTHF research studies, and participant referrals was considered highly successful. An interviewee praised IMARA-SA for its high retention, which the interviewee largely attributed to the participants’ positive experiences in the intervention and through interactions with the study team.

Compared to the highest-scoring feasibility items, scores were lower for having enough time to deliver the intervention (2.2/3.0, SD = 0.9). Echoing these quantitative results, the qualitative data revealed challenges with time management due to the research procedures completed prior to the intervention on Day 1 of the intervention, including enrollment, baseline surveys, and clinical procedures (i.e., HIV/STI testing). Numerous participants recommended separating the research procedures from Day 1 of the intervention because they affected participants’ engagement in the intervention:

*Surveys, HIV testing, STI testing – it was a lot. In terms of the program itself, sitting for so long was a challenge. Because it is long, we would feel the girls and their caregivers would lose interest. By the end, they were so tired.* (Interviewee)

Scores were comparatively lower for perceptions of safety (overall mean = 1.4/3.0, SD = 0.7), including perceived safety of the intervention site (1.3/3.0, SD = 0.9) and travel to/from the site (1.4/3.0, SD = 0.7) – themes which were echoed in the qualitative data. Focus group participants and interviewees, for instance, described the added pressure of completing all the activities on Day 1 before dark, due to safety concerns. Furthermore, the team had to find a workaround for the research procedures since the DTHF clinical team does not come to the site on the weekends, which is when many IMARA-SA sessions were delivered. A clinic team member explained, “*Because of safety, we don’t come in on Saturdays. It’s a very, very unsafe area here.*” The IMARA-SA facilitators and recruiters’ focus groups additionally noted that intervention delivery would be improved if the curricula and workbooks were in Xhosa and English and extra facilitators were trained as back-ups.

### Reach

Scores for the intervention’s reach were high (2.7/3.0, SD = 0.5) (Table 3). In qualitative data, reach was considered one of the project’s greatest successes. Focus group participants and interviewees described very few challenges with enrolling over 640 dyads, particularly after the recruitment procedures were ironed out. A CAB member attributed high reach to the high need for the program offerings: *“It means that IMARA-SA was a necessity.”* Scores were similarly high for reaching the “poorest women” in the mhIST tool (2.8/3.0, SD = 0.5). Indeed, qualitative participants explained that the delivery site and the broad eligibility criteria facilitated access to a population at high risk.

### Adoption

Mean scores for the adoption of IMARA-SA were 3.8/5 (SD = 0.3) (Table 4). IMARA-SA and its clinical procedures were viewed as filling a gap in the services offered in the area by addressing both behavioral and biobehavioral aspects of HIV/STI prevention. Interviewees highlighted the intervention’s family focus as a unique feature compared to other HIV interventions in the area, which have emphasized a biomedical focus in recent years. An interviewee explained, *“The distinguishing or critical factor for IMARA is the involvement of families. IMARA wasn’t looking at a person from an individual perspective but actually from a structural, societal, family perspective.”*

**Table 4.** Ratings of adoption in the full sample and by role

		Total		IMARA-SA study staff (non-facilitators)		IMARA-SA study staff (facilitators)		DTHF staff/ administrator	
#	**Adoption** *(scale of 1-5, where 1=“strongly disagree” and 5=“strongly agree”)*	*n*	Mean (SD)	*n*	Mean (SD)	*n*	Mean (SD)	*n*	Mean (SD)
1	IMARA is more effective than other interventions offering HIV prevention to young women.	27	4.0 (1.0)	18	3.9 (0.9)	5	5.0 (0.0)	4	3.3 (1.5)
2	The IMARA intervention is too complex.*	27	2.9 (1.3)	18	3.2 (1.2)	5	1.6 (1.3)	4	3.5 (0.6)
3	IMARA is successful in preventing HIV/STIs, improving HTC and PrEP uptake, & reducing risk behavior.	27	4.3 (0.8)	18	4.2 (0.8)	5	5.0 (0.0)	4	3.8 (0.5)
4	IMARA is compatible/consistent w/needs of young women in Klipfontein/ Mitchells Plain area.	27	4.4 (0.6)	18	4.4 (0.6)	5	5.0 (0.0)	4	4.0 (0.8)
5	The IMARA intervention requires too many human resources.*	27	2.9 (1.1)	18	2.9 (1.1)	5	2.2 (0.8)	4	3.5 (1.0)
6	IMARA is too expensive (i.e., the costs associated w/ delivering the intervention are too high).*	27	3.5 (1.0)	18	3.7 (0.7)	5	3.0 (1.9)	4	3.5 (1.0)
7	The IMARA intervention is easy to understand and use after training.	27	4.3 (0.7)	18	4.2 (0.8)	5	4.8 (0.4)	4	4.3 (0.5)
8	IMARA has had a visible & substantial impact on young women in Klipfontein/Mitchells Plain	27	4.3 (0.6)	18	4.2 (0.5)	5	5.0 (0.0)	4	4.3 (0.5)
9	Young women are benefitting from the IMARA intervention.	27	4.6 (0.5)	18	4.6 (0.5)	5	5.0 (0.0)	4	4.3 (0.5)
10	IMARA could be easily adapted to fit needs of community organizations that would implement it.	27	4.3 (0.6)	18	4.2 (0.5)	5	5.0 (0.0)	4	4.0 (0)
11	I would only be interested in adopting the IMARA intervention if funding was provided.*	27	2.1 (1.2)	18	2.4 (1.2)	5	1.6 (1.3)	4	1.8 (0.5)
	**Total**	**27**	**3.8 (0.3)**	**18**	**3.8 (0.4)**	**5**	**3.9 (0.4)**	**4**	**3.6 (0.1)**

Abbreviations: IMARA = IMARA-South Africa; STI = sexually transmitted infection, HTC = HIV testing and counselling; PrEP= pre-exposure prophylaxis. Note: Items were adapted from the Antiretroviral Treatment Access Study (ARTAS) [33]. *Indicates that an item was reverse-coded.

The lowest mean scores for adoption concerned the complexity of the intervention (2.9/5, SD = 1.3) and financial implications of the intervention (overall mean = 2.8/5, SD = 0.9), including perceptions that IMARA is too expensive (3.5/5, SD = 1.0), requires too many human resources (2.9/5, SD = 1.1), and that funding would need to be provided for the respondent to adopt the intervention (2.1/5, SD = 1.2). Focus group participants and interviewees suggested that these perceived challenges concerned the complexity and cost of the *research procedures* for the project rather than the intervention itself. They highlighted the challenges with the clinical procedures, which coincided with Day 1 of the intervention, building on the feasibility challenges (described above).

### Sustainability

Mean scores for the sustainability of IMARA-SA were 5.9/7 (SD = 0.8) (Table 5). The highest scores were for the perceived organizational capacity of DTHF, including existing leadership support and organizational systems to support ongoing intervention delivery (items 9–13, mean range = 6.0–6.2). Interviewees explained that DTHF was an ideal site to test the effectiveness of IMARA-SA in a community setting. However, DTHF is a research entity, and thus, the goal was to identify another venue for future delivery and sustainment. Qualitative data revealed numerous possibilities for the future delivery of IMARA-SA, including in schools, clinics, community organizations or NGOs, churches, or community halls. Participants promoted including the clinical procedures as part of the intervention going forward, since the intervention, STI and HIV testing, and provision of PrEP would go *“hand-in-hand”* (interviewee). Participants recognized, however, that integrating clinical testing and PrEP provision would increase the complexity and cost of the intervention (see the adoption section above) and may be less viable in school or community settings compared to clinic settings. Several participants proposed developing a mobile app for IMARA-SA to reach a wider audience.

**Table 5.** Ratings of sustainability in the full sample and by role

		Total		IMARA-SA study staff (non-facilitators)		IMARA-SA study staff (facilitators)		DTHF staff/ administrator	
#	**Sustainability** *(scale of 1-7, where 1=“to little or no extent” and 7=“to a very great extent”)*	*n*	Mean (SD)	*n*	Mean (SD)	*n*	Mean (SD)	*n*	Mean (SD)
1	Advocates/supporters exist who strongly support the IMARA intervention.	27	5.5 (1.3)	18	5.4 (1.4)	5	6.2 (1.1)	4	4.8 (0.5)
2	Advocates or supporters are able to garner resources for the IMARA intervention.	26	5.2 (1.2)	17	5.4 (1.1)	5	5.4 (1.7)	4	4.5 (1.3)
3	The IMARA intervention has leadership support from within the Desmond Tutu Health Foundation.	27	6.3 (1.0)	18	6.2 (1.0)	5	7.0 (0.0)	4	5.8 (1.3)
4	IMARA has leadership support from outside the Desmond Tutu Health Foundation.	24	5.7 (1.3)	16	5.7 (1.2)	5	6.2 (1.3)	3	5.0 (1.7)
5	The IMARA intervention has strong public support.	26	5.9 (1.0)	17	5.9 (1.1)	5	6.6 (0.5)	4	5.3 (0.5)
6	Diverse community organizations are invested in the success of the IMARA intervention.	23	5.4 (1.4)	14	5.2 (1.6)	5	6.4 (0.5)	4	5.0 (0.8)
7	Community leaders are involved with the IMARA intervention.	21	5.5 (1.3)	12	5.3 (1.6)	5	6.2 (0.8)	4	5.0 (0.8)
8	Key stakeholders were engaged in the development of program goals for the IMARA intervention.	25	5.9 (1.2)	16	5.9 (1.3)	5	6.4 (0.5)	4	5.3 (0.5)
9	The IMARA intervention is well integrated into the operations of the Desmond Tutu Health Foundation.	27	6.2 (1.1)	18	6.2 (1.2)	5	6.8 (0.4)	4	5.5 (1.0)
10	Organizational systems are in place to support the IMARA intervention.	25	6.2 (0.9)	16	6.1 (0.9)	5	6.8 (0.4)	4	5.8 (1.0)
11	Leadership effectively articulates the vision of the IMARA intervention to external partners.	23	6.0 (0.9)	14	6.0 (1.0)	5	6.4 (0.5)	4	5.8 (1.0)
12	Leadership efficiently manages staff and other resources for the IMARA intervention.	26	6.2 (1.0)	17	6.1 (1.1)	5	6.8 (0.4)	4	5.8 (1.0)
13	The Desmond Tutu Health Foundation has adequate staff to complete the goals of IMARA intervention.	27	6.0 (1.4)	18	6.0 (1.6)	5	6.0 (1.2)	4	6.3 (1.0)
14	The Desmond Tutu Health Foundation has plans for future resource needs for the IMARA intervention.	22	6.0 (1.0)	14	6.0 (1.0)	5	6.2 (0.8)	3	5.3 (0.6)
15	The Desmond Tutu Health Foundation has a sustainability plan for the IMARA intervention.	21	6.0 (1.2)	14	6.0 (1.3)	4	6.8 (0.5)	3	5.3 (0.6)
16	The goals of the IMARA intervention are understood by all stakeholders.	25	6.0 (1.1)	16	5.9 (1.3)	5	6.6 (0.5)	4	5.8 (0.5)
17	The IMARA intervention clearly outlines the roles and responsibilities for all stakeholders.	24	5.9 (1.2)	15	5.8 (1.4)	5	6.2 (0.8)	4	5.8 (0.5)
	Mean	**27**	**5.9 (0.8)**	**18**	**5.8 (0.9)**	**5**	**6.4 (0.2)**	**4**	**5.4 (0.9)**

Abbreviations: IMARA = IMARA-South Africa. Note: Items were adapted from the Program Sustainability Assessment Tool (PSAT) [34].

Qualitative data also revealed interest in expanding IMARA-SA to other groups, including boys, men, and mixed-gender populations. An interviewee recommended involving the male partners of AGYW because many AGYW participants tested positive for STIs at follow-up even though they were treated at baseline. *“It’s just so hard for our participants to have that discussion with the partner despite all the confidence and building that’s happening between the adolescent and caregiver.”* Some described the importance of including male caregivers to support adolescent girls. As a participant in the CAB focus group explained:

*You get men who play the mother’s role and they do not know how to speak to their daughter about certain things. It will create a conflict. If you can, also include fathers – a father and daughter thing.* (Focus group participant).

Another interviewee expanded on the need to engage men and boys generally:

*We target females for issues around STIs and HIV, but I feel like sometimes we neglect the men. They’re the ones with so much power in the relationships. Maybe if they can also be taught around assertive communication, the importance of using the condom, and not give that responsibility all the time to the women.* (Interviewee).

## Discussion

This study reports the perspectives of study staff, investigators, and collaborators of IMARA-SA, who are well-suited to reflect on the implementation outcomes of interest. We assessed a wide range of implementation outcomes, including reach and sustainability (which are often absent from the literature [20]), from a range of invested groups. We found that IMARA-SA addresses widespread health concerns facing AGYW, a hard-to-reach population. Mean scores were high across outcomes, particularly among IMARA-SA team members (i.e., facilitators and non-facilitators), and study participants discussed the value of the intervention in the community. These findings provide strong support for the implementability of IMARA-SA in the South African context and bode well for the program’s future delivery. We proceed to discuss three key takeaways while addressing the implications of our findings for implementation science researchers and practitioners.

First, our findings highlight tensions between achieving feasibility, delivery site appropriateness, and reach. Safety concerns posed a challenge for feasibility in that staff perceived barriers to participants attending the intervention given concerns about safety – both at the delivery site and in the surrounding area. Yet, at the same time, the delivery site was seen as appropriate and the program’s reach was one of its greatest strengths; by implementing in a community setting, the project was able to meet the population of interest where they live and recruit and retain AGYW living in highly vulnerable areas. Indeed, reaching South African AGYW is a recognized priority due to their vulnerability to HIV [38,39]. These findings have implications for future implementation science researchers in showing how the pursuit of different implementation outcomes might have trade-offs. In this study, the downside of safety concerns was outweighed by the perceived importance of implementing the intervention in a community setting and reaching AGYW and FC most in need. These findings expand on other examples in the literature of the trade-offs that implementation scientists must navigate [40,41] (e.g., between fidelity and adaptation [42]). Indeed, achieving high scores across all implementation outcomes may not be the desired outcome for a project based on contextual realities. By grappling with these trade-offs, implementation science researchers and practitioners can advance not only implementation success but also the science of implementation [40].

Second, our findings illustrate the difficulty of isolating implementation experiences of an intervention when delivered in tandem with extensive research procedures. Compared to the highest scoring items, we observed lower scores for complexity and cost (measures of adoption) and time management (a measure of feasibility). However, the focus groups and interviews revealed that the lower scores on quantitative surveys were primarily a reflection of the research activities and should not be attributed to deficiencies in the intervention itself. Implementation researchers should be aware of the challenges among study participants of distinguishing between intervention and research activities and should strive to find ways of clarifying these distinctions, particularly in surveys. This challenge has been identified by researchers before [43] but is not often addressed in the implementation science literature. These findings clearly illustrate the value of using mixed methods in implementation research; they show how the qualitative data we collected provided a deeper understanding of the topics of interest than would have been achieved with quantitative data alone [26].

Third, our findings provide important insight into the sustainability and future delivery potential of the intervention. Regarding sustainability, DTHF was perceived as compatible for evaluating IMARA-SA in a rigorous clinical trial, with strong organizational skills and mission alignment. Delivering the intervention through DTHF provided valuable insights into the intervention’s delivery potential over time given DTHF’s presence in the community as a trusted biomedical provider. However, DTHF is, by nature, a research entity. Hence, a new implementation avenue is needed to achieve IMARA-SA sustainability. Qualitative findings revealed multiple possible future directions for IMARA-SA, including through schools, clinics, NGOs, churches, or community halls. Most immediately, the intervention has been adapted to a Zambian population through the ZAIMARA project [44], building on the findings presented in this paper [38,45]. While the intervention was perceived as empowering AGYW and their FC, there were suggestions to involve boys and men – and specifically the partners and male caregivers of the AGYW – given attitudes and norms that may impede using learned skills with partners to improve their sexual health. This finding echoes literature highlighting the importance of engaging men and boys in HIV prevention and gender-based violence programming to support women and girls [46–48]. In the U.S., an adaptation of IMARA for Black male caregivers and their girls is currently undergoing assessment [49], which could offer an opportunity for addressing the requests from participants in this study to engage male caregivers.

Study limitations must be noted. Five IMARA-SA staff did not complete follow-up surveys prior to leaving the study. These staff may have reported different implementation experiences than those who remained on the project, but they were not facilitators and only represent a small proportion of the study sample. We did not use the full scales for the implementation outcome measures. While the items we selected generally had acceptable to excellent Cronbach’s alpha, the alpha was lower for feasibility (0.67) and unable to be generated for acceptability given the small sample size. This may have limited the reliability of results for feasibility and acceptability, though this was mitigated in part thanks to the collection of qualitative data which provided a more complete understanding of findings. Additionally, the first author who led the qualitative data collection was the former U.S.-based project director for the pilot work leading up to the RCT, thus closely connected to the project and team and invested in the project’s success. Analytical memoing [27] and extensive discussions within the study team, along with the involvement of a team member external to the SA study team (SA), helped ensure that the findings authentically represent the data [28].

## Conclusions

IMARA-SA staff, investigators, and collaborators reported positive perceptions of the implementation outcomes the intervention. Even in the presence of safety concerns related to delivery site, IMARA-SA was able to reach South African AGYW, a key population vulnerable to HIV. These findings reveal tensions between achieving high reach while maintaining feasibility and site appropriateness due to safety concerns. Researchers must grapple with potential trade-offs in implementing interventions for vulnerable populations. Moreover, our findings illustrate the difficulty of isolating implementation experiences of the intervention itself when delivered in tandem with extensive research procedures; our use of mixed methods proved essential to disentangling feedback on the intervention versus the research procedures. Our findings have already begun to inform future iterations of IMARA-SA and provide valuable insights that can strengthen the implementation of other HIV/STI prevention programs in the region.
